# Should Sex Hygiene Be Taught in the Home and School?*Read at the Austin meeting of the State Society of Social Hygiene, January 27, 1911.

**Published:** 1912-09

**Authors:** Frederick Eby

**Affiliations:** The University of Texas, Austin, Texas


### Should Sex Hygiene be Taught in the Home and School?*

BY DR. FREDERICK EBY,
of the University of Texas, Austin ,Texas.

Since accepting the invitation for this occasion, I have been renewing acquaintance with the subject of sexology by reading hundreds of pages of literature. The melancholy facts which have recently been brought into the limelight by high-minded physicians and other scientists must either degrade the reader into a hopeless pessimism, or impel him to enter the lists as an implacable foe of the social pestilence which today staggers all credulity.

One does not go far without discovering that the entire theme is vastly more complex than anyone has yet dreamed. The social reformers who have been endeavoring to stay the ravages of these unspeakable evils have been ensnared by the physician's fallacy; they have treated the sore and not the system; symptoms and not causes. If results are to be satisfactory, they must follow a more profound study of the problem in its varied aspects. First there must be a higher appreciation of the nature and significance of the sexual life. Then there must be a fuller recognition of the intricate and manifold causes which lead to the widespread, vicious

*Read at the Austin meeting of the State Society of Social Hygiene, January 27, 1911.

prostitution of one of man's noblest functions. The problem of instruction in sex hygiene can not be answered with a simple negation or affirmation.

What is sex, and how does it stand related to man's nature, and to human society? I propose this question because few have yet grasped the grand significance and unparalleled complexity of the sexual life. In consequence, fools rush in with reforms where angels fear to tread. According to President G. Stanley Hall, "sex is the largest and most complex, the most important and interesting of all human themes." To the student of evolution the function of reproduction is as ancient as life itself, reaching back for countless ages to the dawn of all creation. The most primitive forms of cellular matter known to science show clearly the reproductive activity. Because of this antiquity the sexual instinct ranks among the most mechanical or reflex activities of the organism. Ages of exercises have entered into the fixing of the habit of this function. Physiologically, in one aspect at least, it is as mechanical as the pulsations of the heart, or the process of digestion.

The Greeks held the curious idea that Eros, the god of love, was at once the oldest of the gods and the youngest; the first to be born, from whom all others spring, but also the latest. In this beautiful conception they anticipated in myth what we now clearly understand. The propagation of the species is the first but not the only function of sexuality. As a purely animal instinct, reproduction was its function; as an element of human life, it has a vastly deeper and broader import. The animal reproduces an animal like unto itself; man brings forth a being endowed with immortality, a creature who is to be a conscious personality, to think, to suffer and enjoy, to will; a social entity which shall effect for good or ill his fellow creatures. A process must be judged by what it produces and not by its origin. In man, reproduction takes on all the deep solemnity of creation; the impartation of life to beings who have infinite potentialities. Let us away with the vicious notion that it is only an animal instinct for keeping up the race. What is the race to be perpetuated for unless to hand on the splendid moral ideals and characteristics which past generations have accumulated and to which our own shall contribute?

The propagation of the species is only one small aspect of sexuality. Here is where a sad error has crept into our thinking. We have looked at it too narrowly. Throughout the vast periods of development the sexual function has been the greatest agency in producing and fostering the moral qualities which constitute human character, and build our human institutions. Sexuality has imparted masculinity and strength to our bodies. It gave lithesomeness and grace to movement and gesture, and mellowness to voice. The reproductive impulse has been the tap-root of all that is best and noblest in our moral and esthetic life. All the virile traits of men and the distinctive feminine qualities of women are traceable to this source. Courage, endurance, spirit, self-control, generosity; patience, tenderness, and so on, are the fruitage of sex. It has made the home, society, the State, and profoundly influenced the church. "Out of the sex instinct sprang the first altruistic impulses. Around it center the highest aspirations, the noblest sentiments, the loftiest ideals, the purest devotion of which humanity is capable." Sexuality may run its roots into the earth, but it flowers in heaven in the most delicate and beautiful products of human life.

This problem of sex instruction has been thrust upon us not from a new sense of its genetic importance in human culture, but because of the prodigious spread of venereal diseases. In spite of all the attention of learned societies there exists a strange lack of judgment, and diversity of opinion, regarding the causes of the social evil. The leading thinker on the subject on our continent has this to say: "So far as we can apprehend the causes of this evil, the chief determining cause is masculine unchastity, which has it basis in uncontrolled animal instinct, and which is directly encouraged by the double standard of morality for men and women established by social connection." The deeper question comes at once: how are we to explain the phenomena of masculine unchastity and double standard? The causes are numerous and not simple. Society as a whole is sick and not a mere member of it. Let me quite briefly and candidly suggest a few of these underlying causes. I do not mention them in any order of importance, but as they have occurred to me. First there is the tendency to delay marriage. Dr. Prince A. Morrow says: "Marriage, and especially early marriage, is the surest preservative against immorality and its diseases. * * * Every obstacle thrown in the way of marriage is distinctly antisocial and to a certain degree immoral in its tendency." Again, marriage and domestic life have been brought into utter contempt. Domestic infelicity, the cares, and inconveniences, of married life and its lack of independence have been held up to public gaze as a matter of ribald jest. Theater, moving picture show, magazines, newspaper jokes, humorous magazines, and modern plays and novels have conspired to ridicule the sober joys of married life. Over against this they have filled the minds of young men with the notion that the sporty life is independent, smart, exciting. They regard a young man who burdens himself with a wife as selling himself into a life of tiresome monotony, cutting himself from all good times. So long as human society mocks at the married state, and makes it incompatible to support a family, and receive promotion in the world of business, men will prefer Venusberg to the family altar.

The church and the clergy are not entirely blameless. They have systematically ignored the boys and young men, with the inevitable result that these have drifted irrevocably away from religious influences. It is a curious coincidence that the percentage of churchless young men and the percentage of those who are known as the victims of venereal diseases are almost identical. Perhaps at bottom there is here a causal relation. If our religious workers were more engaged in feeding the lambs, and less in fighting extinct theological Satans the social evil would be perceptibly diminished.

There are numerous other causes. The bad posture of boys in crossing the limbs excites abnormal conditions. Very properly did the ancient Greeks rigidly forbid the youth this debilitating habit. Stimulating foods, late hours, the smoking of cigarettes and social excitements have played their rôle in reducing the vitality of immature youth to resist the powerful force of vicious suggestions. Our ideals of womanhood have been degraded. The high cost of living, and the low wages paid to thousands of girls in our cities, have forced them upon the streets to make their living by the unspeakable traffic. Time after time boys used to be approached by news agents in their insinuating manner to induce them to purchase books of the most obscene and lecherous nature. Then we must add to this lewd literature the vicious photographs of nude figures which are secretly circulated among our adolescent youth.

What shall we say of the medical profession? If medical practitioners had been more faithful to their moral responsibilities in speaking the truth and in calling things by their proper names, conditions would not be so lamentable today. Then there is the corrupting example of high society. Bishop Gaylord has been frequently quoted as speaking of "the amazing and unparalleled existence of vice in young men of college and university schools." When it is commonly circulated that rich young vagabonds at the great Eastern colleges keep their mistresses, and still enjoy the leadership in society and college, what will be the effect in undermining the morals of the poorer young fellows?

The silence and dissembling of parents have not been guiltless. As a recent writer says: "It makes infinite difference whether the tone of the school and the home seems to echo and confirm the tone of the street, or whether it counteracts and discredits it." The silence of the home on this one grave matter tends to lead to the impression that in the sphere of sex absolute license is expected.

Finally, alcohol is recognized by all authorities as one of the ugliest predisposing conditions. In one of the shelters for fallen women in New York City during five months 271 girls and women were received. Out of 168 of these, where the information was sufficiently definite to be used for purpose of comparison, 156 were in the habit of using intoxicating liquors, and 121 stated drinking as a single or else partial cause of their downfall. In a large percentage of cases, intoxication was the cause of the initial debauch. The tendency of alcohol to paralyze the higher brain cells in which is lodged the ministry of control, and at the same time its effect in exciting and permanently heightening the activity of the lower centers which are connected with the sexual instinct, are facts of profound significance.

What, then, shall be done by the home and school to ameliorate these fearful conditions and restore sanity and control to the sexual passions? To be candid, students are everywhere in the dark. We are in the condition termed "the disagreement of the inquiring." That something must be done, all consent. Inasmuch as the causes of sexual looseness, as we have seen, permeate the whole structure and organization of society, we can expect no sudden transformation. Success will gradually follow an inner reform of society through a better training of the young and ignorant, through increased facility of marriage and through a loftier ideal of domestic felicity and of womanhood. The more undeveloped and raw an individual is, the more reckless he is in the gratifications of his desires. I wish to offer only a few brief suggestions regarding the course to be pursued by parents and educators.

First of all, let us *normalize* the sex impulse. No other force in all the range of human constitution is so imperious in its powers, so insistent, and so easily excited to the abnormal. We should recognize that this instinct must not be weakened. To enfeeble its virility means to tamper with the underpinning of masculine character; to make sissies of men, and neuters of women. Whatever else we do, we shall be found lined up in a losing battle against nature if we continue to allow higher schools to injure the young by overpressure during adolescence. The stronger the sexual instinct, the better provided it is brought under normal control. The ideal must be to train boys and girls to look upon marriage and procreation and the rearing of a family as the ultimate degree of earthly experience and happiness. Celebacy and race suicide are better than prostitution, but let the truth be emphatically stated, these are not normal. Until we inspire our boys and girls to look forward to parenthood as a divine right and duty we shall fall far short of the best.

Parents must see to it that the sexual life shall not be unduly excited and stimulated. Rich foods, social excitements, tight clothing, the use of cigarettes, bad postures and evil literature must be rigidly avoided. The very best means to prevent the sexual instinct from a morbid condition is vigorous physical exercise, or hard work. All physicians bear witness to this fact. Another prophylactic is a wide circle of intense intellectual interests. The mind of the pubescent youth spontaneously turns to suggestions of sex. It is amazing what paltry things touch off the imagination. No part of our thought so readily passes over into action. It is estimated that 95 per cent of his waking moments is spent by the adolescent in thinking of sexual questions. We must fill his mind with other interests which will draw his attention away, or the results will be disastrous. Secondly, I would lay down this *canon:* The sexual instinct must be *spiritualized.* After all, this is the only real cure for the desperate abuse of man's noblest function. Sexuality is, first, an animal instinct. An instinct is an impulse, an inner force or propulsion. It can be controlled and checked only by some other inner impulse of higher potency. The sexual impulse is the most powerful, and most difficult of all to master. The effort to bring it into subjection trains the most admirable qualities of manhood. For this reason the realm of sexuality has been considered the synonym of morality. Mastery here means strength and mastery everywhere. Sexuality must not be left in the condition of a raw animal instinct. The youth must be led to recognize and value the higher ethical and social relations of this instinct.

It must be *spiritualized;* i. e., made to be the servant of man's will in the realization of the purest and loftiest ideals of manhood. How reformers expect to accomplish anything permanent in this difficult field without invoking the powerful aid of a vital religious experience, I have never been able to conjecture. I am very free to confess that in the final analysis my highest hope lies in the religious development of youth. Not a religion of fear and sentimentality, but a religion of aspiration and longing for the purity and perfection of the Infinite.

One of the most effective forces for the spiritualizing of this instinct is the tendency inherent in the heart of youth to idealize womanhood. The deep reverence of normal youth for mother and womanhood is nature's grand instrument against sexual immorality and vice. In the ancient world the only restraint man acknowledged was prudence. Then followed the period of Christian asceticism, when for the first time in human history man struggled to tame the beast within him. This yearning for mastery over passion was inherently connected with the worship of *virginity*. Later this religious reverence for womanly purity became united with the instinctive chivalry of the Germanic races. In this we behold the building into human character of a Christian attitude toward womankind. We hear frequently that Christianity has elevated woman. It has rather put into the heart of man a new and powerful antidote to his lower life of bestial impulse. The purity of woman has inspired the feeling of worship, her weakness has imparted a noble, manly tenderness, her dependence appealed to man's strength and chivalry, and her beauty and grace have aroused the new sentiment of romantic affection between the sexes. These are the highest aspects of the evolution of the sexual instincts. Where they are carefully fostered the youth possesses a charm which will render him absolutely immune to all the attacks of vicious imaginations from within, or from the vulgar and degrading suggestion from without.

Thirdly, we must *rationalize* the sexual experience. It is absolutely imperative that when children develop in their sexual experience the fuller facts of reproduction be furnished them, so that they shall be able to understand and explain their own instinctive life. But, before stressing this point, let me disabuse your minds of a grand delusion that has fettered the judgment of our reformers. Nowadays we idolize information and instruction. If there is any evil or lack abroad in our land, immediately some one arises to advocate the teaching of the subject in the schools. I am very bold to oppose this foolish delusion. There is a widespread opinion that the youth must be given instruction regarding the frightful dangers which they confront in sowing their wild oats. We are counseled to use the powerful motives of fear and prudence. We must frighten our youth into good conduct. Let me read the opinion of the chief leader in this prophylactic movement in our country. He says: "The only way to make these diseases effective guardians of virtue is to expose their true significance and real danger; to substitute a wholesome fear for the ignorant contempt in which they are now held, the fear of infection, the fear of microbes; to appeal to an enlightened self-interest. After all, fear is the protective genius of the human body, and the basis upon which all hygienic precepts are inculcated." What utter nonsense! Fear is the motive of the weak. Your virile youth scorns your warnings and accepts the challenge. The only fear he respects is the fear of being called a coward. The more you threaten the more he takes the risk to assert his spirit and independence. For several decades we have had in our schools the teaching of temperance physiology. Children are taught the appalling evils that will overtake them if they use alcohol. Has this appeal to fear proved a success? Then why should the amount of liquor consumed in our country increase from year to year?

People expect far too much of moral reform from mere information. Neisser, a German student of this subject, informs us that in certain German cities young men continue to visit houses of prostitution with their pockets full of edifying literature on sex hygiene. If information is the only thing needful, we might expect very superior conduct of medical students. Listen to the great medical authority of sixty years ago, Dr. Wm. B. Carpenter. He says: "There seems to be something in the process of training young men for the medical profession which encourages them in a laxity of principle and of action. It might have been expected that those who are so continually witnessing the melancholy consequence of the violation of the Divine law in this particular would be the last to break it themselves; but this is unfortunately very far from being the case."

Let us have done then with the vain notion, so frequent of late that all we need to do to reform the evils of society is to add a new branch of study to the curricula of the school already groaning with too great a burden. People generally expect too much from mere information and learning. I do not discredit the importance and function of knowledge, but I do emphatically pronounce that there is a vast difference between the filling of the mind with information and the development of the inner moral forces which make for nobility of character, and lead to the control of our passions.

But information there will be inevitably, and rightly. Nothing can hinder it; and this knowledge of the nature of sex has its function in helping the soul, seeking the right, to find and recognize it. How then, where, and by whom shall such information be imparted to the child? Let us not be carried away by idealistic sentimentality, and dream that we can keep the minds of our children innocent of all thought and knowledge of sex. A physician who tested the matter out declared that after several years of searching he has yet to find a boy 12 or 13 who had not false ideas of sex.

Unfortunately, parents, we are not offered the choice of rearing our children in complete innocence of this great theme on the one hand, or of imparting to them pure and scientific knowledge on the other. We are compelled to elect either foul and vicious information, or information that is pure and wholesome. The question for every father and mother is this: shall the minds of my little ones be befouled by the salacious smut of the street, or saved from error by the clean and elevating truth? Shall my child drink pure water, or slime and poison; for drink he must and will.

It is impossible to enter into a discussion of the many intricate problems as to when, and by whom sex information should be given to children. Manifestly a short consideration will convince us that the home is the best place, and the parent the ideal teacher. Much can be done by Sunday school, pastors, Y. M. C. A. workers, and conscientious physicians. But it is obvious that all of these sources are inefficient. This information must unquestionably be furnished in the schools either by physicians, teachers of biology, nature study, domestic science, or morals. The prime problem is that of the materials to be taught. I would briefly propose two conditions: First, the instruction should be progressive, graded to harmonize with the moral and intellectual growth of the child's experience and needs. To furnish this information before the inner checks and controls are developed is to arouse precocious curiosity, to make the sexual appetite morbid, and injure the life. Again, I would insist that at each stage the fullest truth possible shall be given with its proportionate emphasis. Not merely the facts of the physical process of reproduction, but the moral, emotional and spiritual facts as well. The youth must learn of the love of parents for each other, their yearning for children, the undying affection of the mother and father for the child. In the last analysis, the process of reproduction is enveloped in the Eternal mystery of infinite love. He who sees only the physiological incidents is blind as well as vulgar. Let us then teach the full truth to our children.

